# Physicians’ perspectives on adverse drug reactions in pediatric routine care: a survey

**DOI:** 10.1007/s12519-021-00478-1

**Published:** 2021-11-13

**Authors:** Martina P. Neininger, Sarah Jeschke, Lisa M. Kiesel, Thilo Bertsche, Astrid Bertsche

**Affiliations:** 1 Clinical Pharmacy, Institute of Pharmacy, Medical Faculty, Leipzig University and Drug Safety Center, Leipzig University and University Hospital, Bruederstrasse 32, 04103 Leipzig, Germany; 2 University Hospital for Children and Adolescents, Neuropediatrics, Ernst-Heydemann-Str. 8, 18057 Rostock, Germany

**Keywords:** Adverse drug reactions, Pediatrics, Physicians, Routine care, Spontaneous reporting

## Abstract

**Background:**

Children and adolescents are at particular risk for adverse drug reactions (ADRs). We investigated physicians’ perceptions on ADRs in pediatric routine care.

**Methods:**

In this exploratory study from April to November 2020, we invited physicians treating pediatric patients across Germany to complete an online questionnaire consisting mainly of closed questions.

**Results:**

Completion rate was 98% (127/129). Of all participants, 23% (29/127) stated they were not able to estimate how many of their pediatric patients experienced ADRs during drug therapy. The remaining physicians estimated that 7.5% (median; Q25/Q75 3%/20%) of their pediatric patients were affected by ADRs. Regarding counseling on ADRs, 61% (77/127) stated they do not ask regularly the extent to which parents want to be informed. In total, 26% (33/127) stated they avoid counseling on ADRs concerning commonly used approved therapies, whereas only 4% (5/127) did so concerning off-label use (*P* < 0.001). Altogether, 16% (20/127) stated they rather prescribe new medicines as they hope for better effectiveness; 72% (91/127) said they are cautious about doing so owing to yet unknown ADRs. Of all respondents, 46% (58/127) stated they do not report ADRs to the authorities. Concerning the black triangle symbol, a European pharmacovigilance measure, 11% (14/127) stated they knew it and 6% (7/127) stated they reported any suspected ADR for drugs with that symbol.

**Conclusions:**

Physicians’ perspectives on ADRs were ambivalent: ADRs influenced their parent counseling and drug prescribing; yet, they struggled to estimate the impact of ADRs on their patients and were not aware of specific pharmacovigilance measures.

**Supplementary Information:**

The online version contains supplementary material available at 10.1007/s12519-021-00478-1.

## Introduction

Children and adolescents are particularly vulnerable to adverse drug reactions (ADRs). ADRs are more frequent and can be more severe in children than in adults [1, 2]. Reliable data on ADRs in children and adolescents are very limited [3], especially due to the frequent off-label use. A review on drug prescribing in pediatric routine care showed that as many as 65% of prescriptions in the hospital setting and 31% of prescriptions in the primary health care setting were off-label [4]. These represent a risk in the treatment of children and adolescents. Thus, spontaneous reports are an important source of information to better characterize the risks of drugs in pediatric patients [5]. However, a review showed under-reporting of ADRs [6]. For example, in Germany, the reporting rate in children is only half as high as in adults [7]. This discrepancy could be caused by the fear of potential legal consequences in the case of ADRs owing to medication errors or off-label use [8]. Studies showed a major impact of ADRs on the quality of life of children/adolescents and their families [9–11] and on therapy failure due to ADRs [12]. However, little is known about the extent to which physicians include ADRs in their treatment decisions or in the communication to parents and patients. The objective of our study was to investigate the physicians’ perspectives on ADRs in the routine care of children and adolescents.

## Methods

### Study design

In this exploratory study, the target population comprised physicians treating pediatric patients in primary or hospital care. We invited the participants to complete an online questionnaire. The intended sample size was 125 participants selected by convenience sampling. The survey was conducted from 29 April 2020 to 12 November 2020.

### Ethic approval and informed consent

The local ethics committee granted approval for this study. The participant information explained that completion of the questionnaire took about 10 minutes; participation in the survey was voluntary; data assessment was anonymous; who the investigator was; and the purpose of the study. Further, the physicians were informed that by submitting the questionnaire they gave informed consent to participate in the study.

### Questionnaire

The questionnaire was based on researches on patients’ perceptions on adverse drug reactions [9, 10]. In this research, some points remained open that can only be answered by considering the physician’s perspective. In a qualitative survey among physicians, challenges in drug reporting were detected [13]. However, data on the reporting behavior by the physicians themselves are scarce. Thus, we aimed to close those gaps.

We developed a questionnaire consisting mainly of questions with pre-set answers to tick, Likert scales to express the level of agreement with a given statement, or numbers to fill in (Online Resource 1). In one question, the participants were asked to prioritize the three answers most relevant to them. The questionnaire aimed to capture the physicians’ perceptions and experiences and addressed the following issues: occurrence and consequences of ADRs in routine care, causes for ADRs, measures to be taken in the case an ADR occurs, impact of ADRs on parent/patient counseling and drug prescribing, and ADR reporting. In addition, one question referred to the black triangle in the context of ADR reporting. The black triangle is part of a comprehensive concept to enhance pharmacovigilance in the European Union [14]. It indicates “This medicinal product is subject to additional monitoring” and is printed in the package leaflets and summaries of product characteristics of the respective medicinal products since 2013. For medicinal products marked with the black triangle, any suspected ADR should be reported to the authorities.

The questionnaire was pre-tested in a pilot survey with four physicians working in pediatric care and one pharmacist experienced in ADR assessment in pediatric patients. Based on the pilot survey, we optimized the questionnaire regarding comprehensibility, clarity, and readability. We also tested and optimized usability and technical functionality.

We did not use randomization of items. The questionnaire was distributed over two pages. The first page contained the questions on ADRs (15 items), the second page dealt with sociodemographic data (6 items). The participants were able to review and change their answers through a back button.

### Recruitment

We distributed the invitation to participate in the open online survey through announcements on the website of the University Hospital for Children and Adolescents, Neuropediatrics, Rostock, Germany; e-mails to physicians who had previously referred patients to the university hospitals for children and adolescents of Rostock and Leipzig, Germany; and through websites or mailing lists of the following medical associations: DGKJ (German Society of Pediatrics and Adolescent Medicine), STGKJM (Saxon-Thuringian Society for Pediatrics, Adolescent Medicine and Pediatric Surgery), SGKJ (South German Society for Pediatrics and Adolescent Medicine), social pediatric centers’ mailing list of the DGPSJ (German Society for Social Pediatrics), and BVKJ (German Professional Association of Pediatricians). No incentives were offered to the participants.

### Survey administration

The survey was accessible without registration on the website of the University Hospital for Children and Adolescents, Neuropediatrics, Rostock, Germany. The link provided in the invitation mails and newsletters led directly to the questionnaire. We collected data using Evasys, a software for conducting surveys. With this software, participants use a web browser to enter data into the questionnaire. The resulting data are stored in a database, compliant with European data protection guidelines. These data can be transferred from the database to an Excel file, from which an SPSS file can be generated. Since participation was anonymous, we did not use mechanisms to determine a unique visitor, such as IP check or log file analysis in compliance with European data protection guidelines. Since no personal information was collected, no specific mechanisms to protect unauthorized access were necessary. The digital data were archived on a password-protected external hard drive, which is stored under lock and key accessible only to authorized persons.

To prevent the assessment of double entries from the same user, we analyzed the sociodemographic data for duplications. In case of a duplication, the first entry was used. We calculated the completion rate as ratio of participants who finished the survey divided by the number of participants who agreed to participate. We included only questionnaires in the analysis in which at least 50% of the items were filled in. We also calculated the completeness rate as a measure of how complete the questionnaires were filled in. Since it was not mandatory for the participants to answer all the questions, we did not use a completeness check before questionnaire submission.

### Statistical analysis

To compare data, we used a Wilcoxon signed-rank test for paired data and a Mann–Whitney *U* test or Chi-squared tests for unpaired data. A *P* value ≤ 0.05 was considered to indicate significance. Statistical analysis was conducted using IBM SPSS Statistics Version 26 (IBM Corporation, Armonk, New York, USA). To express the ranking of the prioritization, we calculated a score (*S*) reflecting the prioritization and frequency of each answer: *S* = 3*n*_1_ + 2*n*_2_ + *n*_3_; in this context, *n*_1/2/3_ indicates the number of nominations of the respective answer at position 1/2/3. We did not perform any statistical corrections, such as weighting of items or propensity scores.

## Results

### Participants

Of 129 physicians who agreed to participate, 127 finished the survey resulting in a completion rate of 98%. In 80% (102/127) of the questionnaires, all items were filled in. In 16% (20/127) of the questionnaires, 95% of questions were answered, and in 4% (5/127) of the questionnaires, the completeness rates were between 70% and 90%. In all questionnaires, the completeness rate was higher than 50%, and no duplicates were identified in the assessment of the sociodemographic data; thus, we included all questionnaires in the further analyses. Participants’ characteristics are shown in Table 1.

**Table 1:** Participants’ characteristics

Parameters	Values
Total number [*n*] (male/female/not stated)	127 (50/73/4)
Median age (Q25/Q75; min./max.) (y)	50 (39/57; 27/76)
Median professional experience (Q25/Q75; min./max.) (y)	22 (10/30; 0.8/44)
Specialization [*n* (%)]	
Pediatrics	115 (91)
	thereof 13 in pediatric training
	thereof 34 working in neuro-/social pediatrics
Child and adolescent psychiatry	5 (4)
General medicine	3 (2)
Other specialization or not specified	4 (3)
Work setting [*n* (%)]	
Ambulatory	54 (43)
Inpatient	16 (13)
Ambulatory and inpatient	56 (44)
Not specified	1 (1)
Localization [*n* (%)]	
Rural community (< 5000 inhabitants)	3 (2)
Small town (5000 to < 20,000 inhabitants)	16 (13)
Medium-sized town (20,000 to < 100,000 inhabitants)	35 (28)
City (≥ 100,000 inhabitants)	73 (57)

### ADRs in physicians’ routine care

Of the 127 physicians, 69 (54%) said that ADRs play a minor role in their routine care, and 4 (3%) stated that they play no role at all. In contrast, for 47 (37%) of the respondents, ADRs play a moderate role in their routine care, and for 7 (6%) a major role.

Of all 127 participants, 29 (23%) stated they were unable to estimate how many of their pediatric patients experienced ADRs during drug therapy. The other physicians estimated that 7.5% (median; Q25/Q75: 3%/20%) of their pediatric patients were affected by ADRs. Of the 127 physicians, 42 (33%) felt unable to estimate how many patients needed an ambulatory treatment due to ADRs. The remaining participants estimated that 2% (median; Q25/Q75 1%/5%) of their pediatric patients needed an ambulatory treatment due to ADRs. Regarding the frequency of hospitalization or a prolonged hospital stay due to ADRs, 49 (39%) of the 127 physicians could not estimate how many of their pediatric patients were affected. Of all 127 respondents, 11 (9%) stated that none of their pediatric patients had to be hospitalized or experienced a prolonged hospital stay due to ADRs. The remaining physicians estimated that 1% (median; Q25/Q75: 0.5%/2.5%) of their pediatric patients were affected by (prolonged) hospitalization. The professional experience of these physicians (median: 22.5 years; Q25/Q75: 13/30 years) did not differ from those (median: 23 years; Q25/Q75: 8/32 years; n.s.) who said none of their pediatric patients had to be hospitalized or experienced a prolonged hospital stay.

### Causes of ADRs

In a question concerning the most important causes of ADR with multiple answers possible, 82 (65%) of the 127 participants chose “profile of the active ingredient”, 44 (35%) “disposition (genetic/underlying disease”, 40 (31%) “dosage errors”, 40 (31%) “dosage increase”, 33 (26%) “interactions between active ingredients”, and 25 (20%) “non-adherence of the patient/parents”. Physicians working in neuro-/social pediatrics mentioned “dosage increase” ([23/34 (68%) vs. 17/91 (19%), *P* < 0.001)] and “interactions between active ingredients” [16/34 (47%) vs. 17/91 (19%), *P* = 0.001] more frequently than other participants. Single mentions on other causes referred to parents’ concerns triggered by the information about possible ADRs and a general ambivalence of the parents concerning drug administration to their children (*n* = 3/127, 2%), allergies, use of drugs in the wrong indication (e.g., exanthema due to amoxicillin in infectious mononucleosis), or exogenous factors (each *n* = 2/127, 2%). Of all 127 participants, 8 (6%) felt unable to assess the most important causes for ADRs.

### Measures taken in case of an ADR

**Fig. 1:**
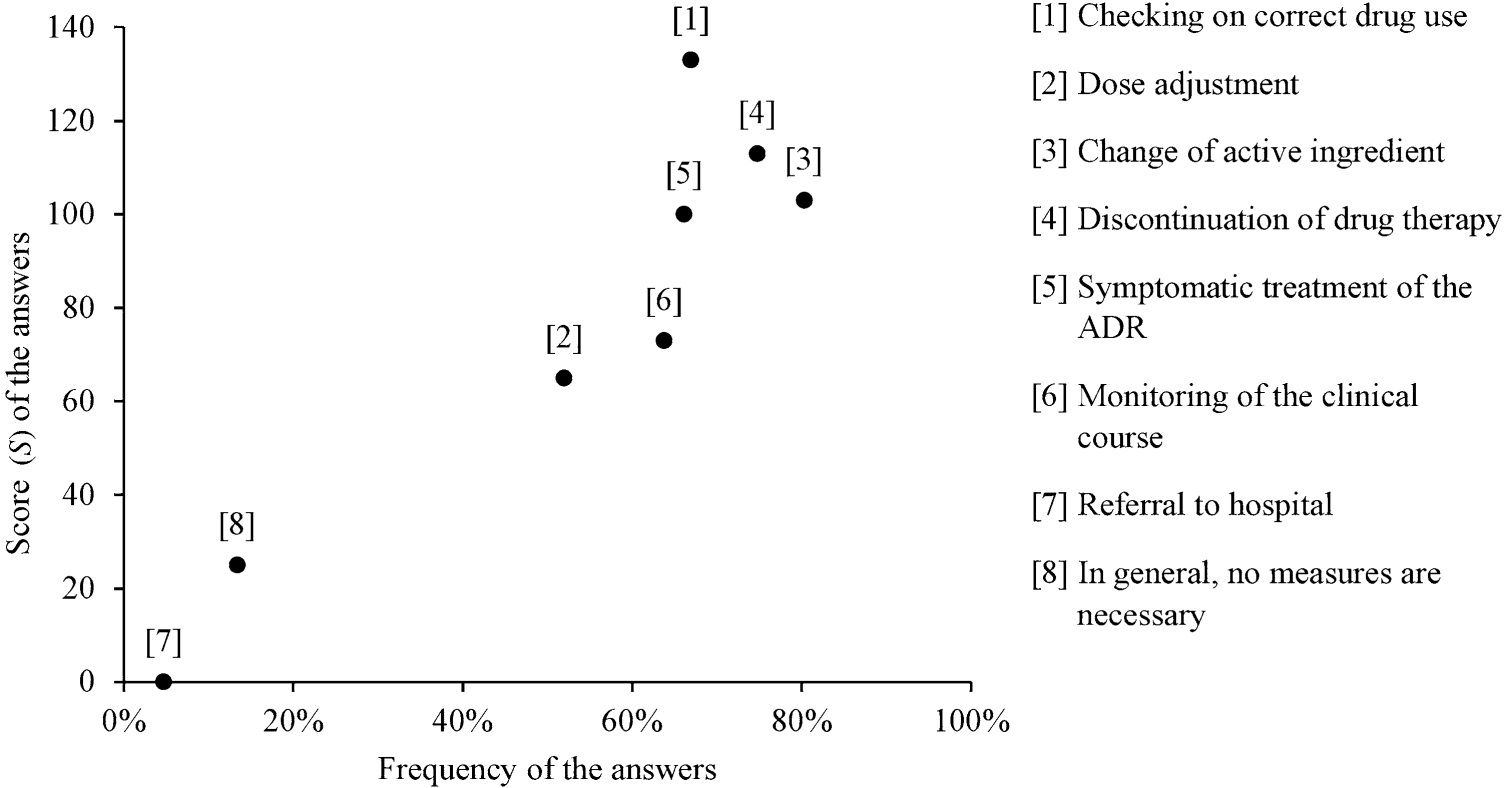
Physicians’ most frequent measures when adverse drug reactions occur in pediatric patients (multiple answers possible). The score (*S*) was calculated from the physicians’ prioritization of the answers and frequency of each answer: *S* = 3*n*_1_ + 2*n*_2_ + *n*_3_; in this context, *n*_1/2/3_ indicates the number of nominations of the respective answer at position 1/2/3

The participants’ prioritization of the most frequent measures taken in case of an ADR is shown in Fig. 1. The highest priority was given to “checking on correct drug use”, “discontinuation of drug therapy”, “change of the active ingredient”, and “symptomatic treatment of the ADR” (Fig. 1). Of all 127 respondents, 16 (13%) felt unable to prioritize their answers. Some physicians added further statements on measures taken in case of an ADR: 3 (2%) referred to talking with the parents, e.g., “Discussion of the next steps with patients and parents, explain with patience, participatory decision-making.”;  1 (1%) participant mentioned the “use of supportive medication e.g. to protect the intestinal flora when antibiotics are administered”; and 2 (2%) respondents said they cannot give a statement on frequent measures taken in case of an ADR.

### Parent counseling on ADRs

**Fig. 2:**
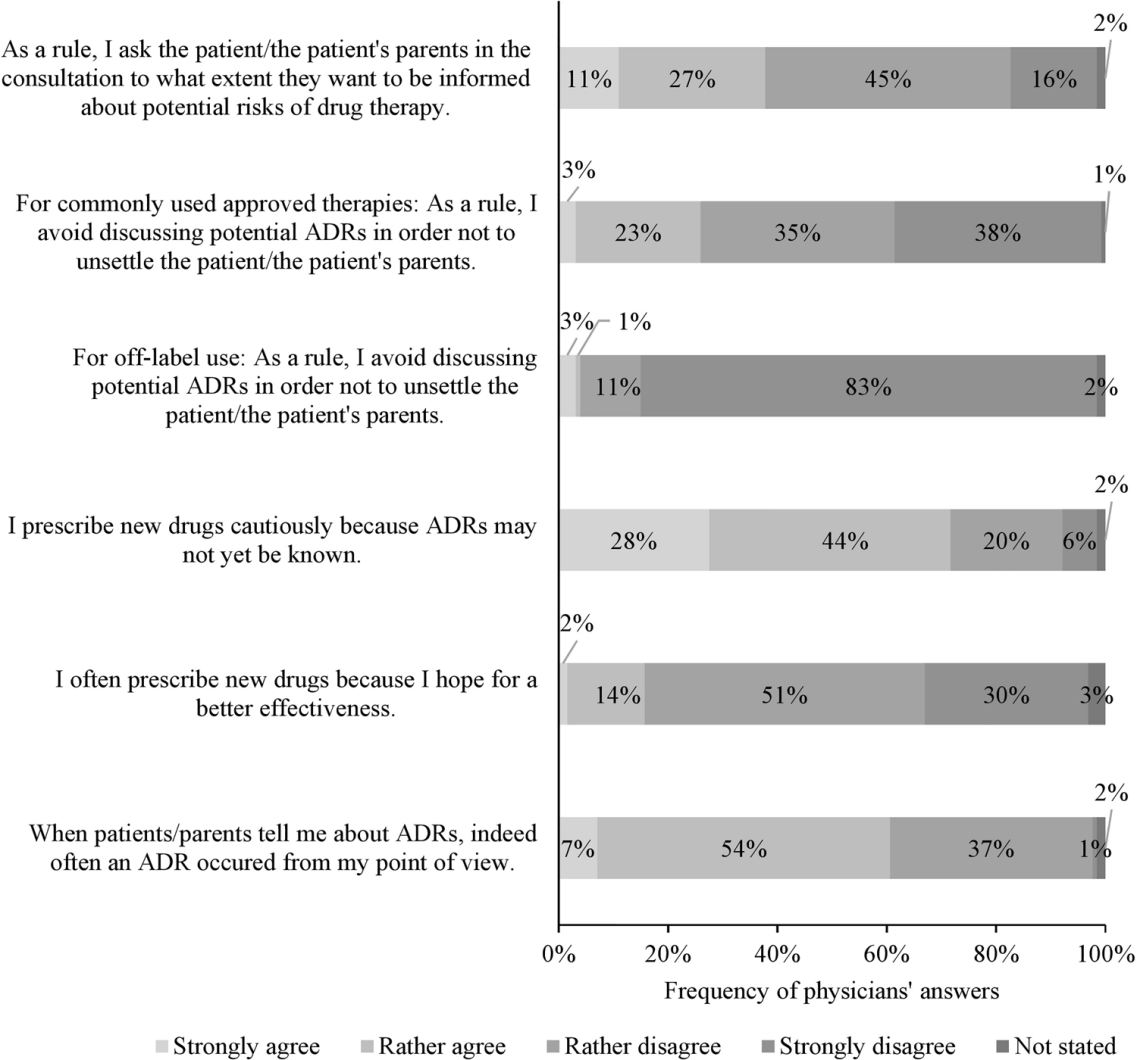
Physicians’ level of agreement with given statements on drug prescription and parent counseling. *ADR* adverse drug reaction

The physicians’ agreement with statements on parent counseling is shown in Fig. 2. Of the 127 participants, 77 (61%) disagreed with the statement “As a rule, I ask the patient/the patient's parents in the consultation to what extent they want to be informed about potential risks of drug therapy”. The physicians clearly distinguished in their counseling between commonly used approved therapies and off-label use (*P* < 0.001): of the 127 participants, 33 (26%) stated they avoid counseling on ADRs concerning commonly used approved therapies in order not to unsettle the patient/the patient's parents. Concerning off-label use, 5/127 (4%) said they avoid counseling on ADRs. Of the 127 respondents, 77 (61%) stated that from their point of view in most cases actually an ADR occurred when the parents report about it. Some participants felt the desire to make further statements: “I always inform about relevant and frequent and severe ADRs, regardless of the wish of the family.”; “For all those who complain about ADRs, a dialogue must be sought and solutions found.”

### Influence of ADRs on physicians’ prescriptions

Of the 127 physicians, 91 (72%) stated they prescribe new drug cautiously because ADRs may not yet be known; 20/127 (16%) stated they frequently prescribe new drugs because they hope for a better effectiveness (Fig. 2).

### Physicians’ experiences with ADR reporting

With regard to ADR reporting, 58 (46%) of all 127 participants stated they do not report to the responsible authorities at all. The remaining physicians stated they reported ADRs in median once (Q25/Q75: 1/1.625 times) a year. Two respondents added further statements such as “Less than once per year. I used to do this more often, but it always meant a lot of work for me and a lot of questionnaires to fill out.”; “Very rarely. I reported in some cases and it always resulted in so many follow-up questions that I have become cautious.” On the question “Do you know the black triangle symbol”, 14 (11%) of all 127 participants stated “yes”; 7 (6%) of all 127 respondents stated they report any suspected ADR to the authorities for drugs labeled with that symbol.

## Discussion

In our exploratory study on physicians’ perspectives on ADRs in pediatric patients, we found that more than half of the participating physicians said that ADRs are of minor importance in their routine care. However, they stated that ADRs have an impact on their parent and patient counseling and on their therapy decisions. Many participants stated that they do not report ADRs to authorities and were not aware of the black triangle, a pharmacovigilance measure in the European Union.

### Relevance of ADRs in physicians’ routine care

In our study, a considerable number of physicians felt unable to estimate how many of their pediatric patients experienced ADRs or needed ambulatory/inpatient treatment due to ADRs. This could indicate that physicians have a limited awareness of ADRs in their patient care. Literature on the population rate of ADRs in children is scarce. A study conducted in six office practices in Boston, USA reported a prevalence of 16% children who experienced ADRs in ambulatory care [15]. In our study, those physicians who gave an estimate rated a much lower prevalence. In addition, almost 10% of the physicians assumed that none of their pediatric patients had to be hospitalized due to ADRs. This does not seem realistic considering the results of various studies on the causes for hospitalization of children: one study reported 2.9% of hospital admissions to be due to ADRs in the UK [16]. A review showed that a median of 2.85% of pediatric visits in an emergency department were due to ADRs [17]. In a US-wide study on children attending the emergency department because of ADRs, 9.5% of the children had to be hospitalized [18]. In children aged younger than 1 year, ADRs were the third most common cause for non-fatal injuries leading to an emergency department visit [18]. Altogether, the physicians in our study potentially underestimated the occurrence of ADRs and seem to have an ambiguous attitude towards the relevance of ADRs: the vast majority said that ADRs do not play a major role for them in patient care. At the same time, the majority stated that they do not use new drugs because of the potential for unknown ADRs.

### Causes for ADRs

The cause for ADRs most frequently chosen was unsurprisingly “profile of the active ingredient”. Interestingly, about one-third of the participants chose each “disposition [genetic/ underlying disease]”, “dosage errors”, “dosage increase”, and one-quarter “interactions between active ingredients” as important causes for ADRs in children. ADRs arising from the profile of the active ingredient and the patient’s disposition can hardly be influenced. For some drugs, dose adjustment can lead to a reduction of those ADRs. If dose adjustment is not possible, the only alternative is the choice of another active ingredient. Thus, those ADRs are frequently non-preventable if no appropriate alternative drug exists. In contrast, dosage errors are preventable. Unintentional overdoses were reported to account for almost half of ADRs leading to an emergency department visit [18]. Another study on treatment of pediatric patients by paramedic emergency medical services reported up to 36% of patients being affected by dosage errors [19]. Among other reasons, dosage errors can be caused either by wrong physician’s calculation, miscommunication to the patients/parents, or wrong patients’/parents’ usage of the drug [20–24]. Consequently, “checking on correct drug use” was the measure to be taken in case of an ADR with the highest priority according to the physicians. Established methods to avoid dosage errors are calculation support, e.g., clinical decision support systems, written information including pictograms for the patients/parents, appropriate dosing tools, and practical training of the parents in drug administration [20–23]. Physicians should make every effort to provide patients and their parents with appropriate counseling on drug use, tailored to their needs.

### Measures in case of ADRs

“Change of active ingredient” and “discontinuation of drug therapy” were classified as most important measures in case of an ADR by the physicians. If we take again a closer look on possible causes for ADRs, “dosage increase” and “interactions” were chosen mainly by neuropediatricians shedding a light on a specific patient group. Those patients frequently receive anti-seizure medications. It has been shown that anti-seizure medications commonly used in pediatrics, such as valproate, oxcarbazepine, or levetiracetam, frequently fail owing to limited therapeutic effectiveness or ADRs leading to modifications in drug therapy or discontinuation of an active ingredient [12]. The occurrence of these events can only be avoided to some extent, as the effectiveness can only be influenced slightly. Approximately one-third of patients do not become seizure-free even with long-term anti-seizure medication [25]. With regard to ADRs, it is important before treatment initiation to consider which ADRs may occur and to choose the anti-seizure medication that best meets the needs of the patients. For example, psychiatric and neurologic ADRs were judged by pediatric patients and their parents to negatively affect peer interaction and school/leisure activities [9, 10]. Paying more attention to these patient-specific perceptions when selecting drug therapy might help to prevent discontinuation of drug therapy or change of active ingredients.

### Communication on ADRs

Every fifth participant chose “Non-adherence of the patients/parents” to be a relevant cause for ADRs. If we take a closer look at possible reasons for non-adherence, several findings of our study imply barriers in the communication about ADRs and the parents’ individual needs are not always sufficiently met. For example, physicians avoided talking about ADRs in order not to unsettle the patient/the patient’s parents or did not ask to what extent the parents want to be informed about the potential risks of the drug therapy. Parents have different desires on how potential ADRs are communicated to them [10]. Thus, it is important that physicians take the parents’ and patients’ concerns seriously and provide advice to alleviate those concerns, offer support and give further explanations, e.g., on the importance of regular drug intake [9, 10, 26]. In addition, children and adolescents with chronic conditions desire to be actively involved in physicians’ counseling [9, 26, 27]. Those measures also could contribute to increase patients’ adherence [28–30].

### Physicians’ experiences with ADR reporting

Almost half of the participants stated they do not report ADRs to the responsible authorities. This means that these participants also do not report unexpected or serious ADRs. It remains unclear whether no ADRs have occurred so far, whether ADRs that had occurred were not recognized, or whether ADRs that had occurred were recognized and yet not reported. The remaining participants stated they report ADRs in median once a year. This underlines other findings that ADRs in pediatrics are underreported [31]. Two participants felt the need to explain that the reporting procedure is too burdensome and that they have had unfavorable experiences with it in the past. A qualitative study among physicians identified an additional amount of work as a barrier to ADR reporting [13]. Barriers to effective pharmacovigilance become also obvious in our study as almost 90% of participants stated they did not know the black triangle at all. This indicates that knowledge about this pharmacovigilance measure is not widespread. In our opinion, a rather practical barrier exists: physicians only consult the summary of product characteristics when they need in-depth information; if they have the needed information available, they do not look at it and, thus, do not notice the symbol. As a possible solution, the practice information system could give an alert when an affected medicinal product is prescribed.

Spontaneous reports or case reports by health care professionals were identified as the most important information source for drug safety issues leading to withdrawal of drug approval [32]. It has been shown in long-term studies that about 20% of approved new drugs acquired black box warnings or were withdrawn due to safety issues [33, 34]. An Italian study showed that 10% of the reported ADRs affecting pediatric patients were not described in the summary of product characteristics, and 8% were related to off-label use [5]. It is, therefore, essential to generate data from routine pediatric care to allow a better assessment of the benefit-risk ratio of drugs in pediatric patients [35]. The general importance of their contribution to pharmacovigilance should be better communicated to physicians, e.g., in advanced training [31]. The resulting positive impact on patient safety should be emphasized to physicians so that ADR reporting does not remain an abstract administrative task. In addition, the reporting procedure should be simplified in order not to discourage physicians due to a high bureaucratic effort. For example, rather than creating a report for every single suspected ADR, physicians could create a quarterly report containing all ADRs for the respective drug. This might improve reporting frequency, and thus provide a more realistic assessment of ADRs.

### Limitations

Physicians interested in the topic of ADRs were more likely to participate in this study. Thus, it can be assumed that in general ADRs are an even minor issue in physicians’ routine care and that an even lower percentage of physicians engages in ADR reporting. As this was an online survey in which responses depend primarily on honesty and are partly affected by the recall ability, there could be a chance of bias. A reliability testing of the questionnaire was not performed, which may limit the consistency of the results.

In conclusion, physicians’ perspectives on ADRs were ambivalent: on the one hand, ADRs influenced their parent counseling and choice of drug therapy. On the other hand, they had problems to estimate the impact of ADRs on their patients. Physicians report ADRs rather seldomly to the authorities, resulting in a potential gap in patient safety. To increase general patient safety in pediatrics, physicians’ vigilance should be enhanced. Pharmacovigilance measures should be better communicated to physicians, and the reporting processes should be simplified to fit better in the physicians’ routine care.

## Supplementary Information

Below is the link to the electronic supplementary material.

Supplementary file1 (PDF 78 KB)

## Data Availability

The datasets generated during and/or analyzed during the current study are available from the corresponding author on reasonable request.
