# Supplementary material for: Physicians’ perspectives on adverse drug reactions in pediatric routine care: a survey
Source: World J Pediatr. 2021 Nov 13;18(1):50–8. doi: 10.1007/s12519-021-00478-1 (PMC8761136; doi:10.1007/s12519-021-00478-1)
Supplement: Supplementary file 1 — Supplementary file1 (PDF 78 KB) [file 12519_2021_478_MOESM1_ESM.pdf]

Supplemental Material

Article title: Physicians' perspectives on adverse drug reactions in pediatric routine care – a survey

Journal: World Journal of Pediatrics

Online Resource 1 Questionnaire

The original questionnaire was conducted in German. For a better understanding of the survey, we translated the original questionnaire. ADRs: adverse drug reactions

Question	Answer
What role do ADRs play in your routine care?	[1] Major role<br>[2] Moderate role<br>[3] Minor role<br>[4] No role at all
Please estimate how many of your pediatric patients experience ADRs during drug therapy.	About ______%<br>○ I cannot assess that.
Please estimate how many of your pediatric patients need to be treated as a result of an ADR on an outpatient basis (e.g. in your practice or on an outpatient basis in the hospital).	About ______%<br>○ I cannot assess that.
Please estimate how many of your pediatric patients need inpatient hospital treatment as a result of an ADR, or how many have a prolonged inpatient stay.	About ______%<br>○ I cannot assess that.
In your opinion, what are the main causes for the occurrence of ADRs in pediatric patients? (Multiple answers possible)	[1] Profile of the active ingredient<br>[2] Dosage increase<br>[3] Interactions between active ingredients<br>[3] Dosage errors

	[4] Non-adherence of the patient/parents<br>[5] Disposition [genetic/ underlying disease]<br>[6] Other____________<br>[7] I cannot assess that.
Which measures are the most frequent reactions in your routine care in case of an ADR in your pediatric patients? (Multiple answers possible)<br><br>Please prioritize the 3 most important measures.	[1] Checking on correct drug use<br>[2] Dose adjustment<br>[3] Change of active ingredient<br>[4] Discontinuation of drug therapy<br>[5] Symptomatic treatment of the ADR<br>[6] Monitoring of the clinical course<br>[7] Referral to hospitalization<br>[8] In general, no measures are necessary<br>[9] Other___________<br>[10] I cannot assess that.
Please position yourself on the following statements:<br>„As a rule, I ask the patient/the patient's parents in the consultation to what extent they want to be informed about potential risks of drug therapy.“<br>„For commonly used approved therapies: As a rule, I avoid discussing	For each statement:<br><br>[1] Strongly agree<br>[2] Rather agree<br>[3] Rather disagree<br>[4] Strongly disagree

potential ADRs in order not to unsettle the patient/the patient's parents." „For off-label use: As a rule, I avoid discussing potential ADRs in order not to unsettle the patient/the patient's parents." „I prescribe new drugs cautiously because ADRs may not yet be known." „I often prescribe new drugs because I hope for a better effectiveness." „From my point of view when patients/parents tell me about ADRs, indeed often an ADR occurred."	
How frequently do you report ADRs approximately per year to the responsible authorities?	About _________ reports per year.<br>○ I don't have the time to report in my routine care.
Do you know the black triangle in the context of ADR reporting?	[1] Yes<br>[2] No
Which ADRs do you report for drugs marked with the black triangle? (Multiple answers possible)	[1] Expected<br>[2] Unexpected<br>[3] Slight<br>[4] Severe<br>[5] Frequent<br>[6] Rare<br>[7] All

Sociodemographic data	
Sex	[1] Male
	[2] Female
	[3] Divers
Age	____years
Professional experience	____years
Specialization	[1] ____________{Free answer}
	[2] In training for the following specialization ______________
Work setting	[1] Ambulatory
	[2] Inpatient
	[3] Ambulatory and inpatient
Localization	[1] Rural community (<5,000 inhabitants)
	[2] Small town (5,000 - <20,000 inhabitants)
	[3] Medium-sized town (20,000 - <100,000 inhabitants)
	[4] City (≥100,000 inhabitants)
